# Improved therapy for neuroblastoma using a combination approach: superior efficacy with vismodegib and topotecan

**DOI:** 10.18632/oncotarget.7714

**Published:** 2016-02-25

**Authors:** Nagendra K. Chaturvedi, Timothy R. McGuire, Don W. Coulter, Ashima Shukla, Erin M. McIntyre, John Graham Sharp, Shantaram S. Joshi

**Affiliations:** ^1^ Departments of Genetics, Cell Biology and Anatomy, University of Nebraska Medical Center, Omaha, NE, USA; ^2^ Departments of Pharmacy Practice, University of Nebraska Medical Center, Omaha, NE, USA; ^3^ Departments of Pediatrics, Division of Pediatric Hematology and Oncology, University of Nebraska Medical Center, Omaha, NE, USA

**Keywords:** neuroblastoma, small molecule inhibitors, MYCN, hedgehog inhibitor, chemotherapy

## Abstract

Aberrant activation/expression of pathways/molecules including NF-kB, mTOR, hedgehog and polo-like-kinase-1 (PLK1) are correlated with poor-prognosis neuroblastoma. Therefore, to identify a most efficacious treatment for neuroblastoma, we investigated the efficacy of NF-kB/mTOR dual-inhibitor 13-197, hedgehog inhibitor vismodegib and PLK1 inhibitor BI2536 alone or combined with topotecan against high-risk neuroblastoma. The *in vitro* efficacy of the inhibitors alone or combined with topotecan on cell growth/apoptosis and molecular mechanism(s) were investigated. Results showed that as single agents 13-197, BI2536 and vismodegib significantly decreased neuroblastoma cell growth and induced apoptosis by targeting associated pathways/molecules. In combination with topotecan, 13-197 did not show significant additive/synergistic effects against neuroblastoma. However, BI2536 or vismodegib further significantly decreased neuroblastoma cell growth/survival. These results clearly showed that vismodegib combination with topotecan was synergistic and more efficacious compared with BI2536 in combination. Together, *in vitro* data demonstrated that vismodegib was most efficacious in potentiating topotecan-induced antineuroblastoma effects. Therefore, we tested the combined efficacy of vismodegib and topotecan against neuroblastoma *in vivo* using NSG mice. This resulted in significantly (p<0.001) reduced tumor growth and increased survival of mice. Together, the combination of vismodegib and topotecan showed a significant enhanced antineuroblastoma efficacy by targeting associated pathways/molecules which warrants further preclinical evaluation for translation to the clinic.

## INTRODUCTION

Neuroblastoma, the most common pediatric malignancy of neural crest origin, accounts for 8-10% of childhood cancers and 15% of pediatric cancer deaths. The clinical outcomes of patients with neuroblastoma are highly variable, ranging from spontaneous regression to fatal progression of the disease [1, 2]. On the other hand, approximately 60% of patients are diagnosed as high risk with metastases and these patients have remained as a therapeutic challenge for pediatric oncologists. Despite intensive multimodal therapy including radiation, surgery, and chemotherapy, the overall survival of high risk patients has remained at less than 40% [3]. Amplification of the MYCN gene, which occurs in 40-50% of the high risk neuroblastoma cases, remains the major key predictor of poor outcomes. Patients with high risk neuroblastoma and those with MYCN amplification typically show emergence of treatment resistance [3–5]. Thus, novel effective therapies are urgently needed to improve clinical outcomes of high risk neuroblastoma patients.

Since therapy resistance appears to be a key feature of many tumors, including high risk neuroblastoma, identification of underlying mechanisms and pathways that play crucial roles in neuroblastoma cell proliferation and drug resistant survival, could substantially enhance the development of therapies for this tumor and other forms of cancer [5]. Evidence suggests that certain oncogenic pathways and cell cycle regulators such as NF-kB, PI3K/mTOR, hedgehog and polo-like kinase 1 (PLK1) are overexpressed and activated in high risk neuroblastoma [6–15]. Aberrant expression and activation of these pathways/molecules have not only been associated with many aspects of neuroblastoma tumorigenesis, but also in chemoresistance of the disease [16–19]. Therefore, these pathways/molecules are viable and logical targets for the treatment of refractory neuroblastoma. To that end, a number of small molecule inhibitors targeting these key pathways and molecules have recently been developed [20, 21].

Recently, a quinoxaline analog 13-197, a NF-kB and mTOR dual small molecule inhibitor, has been described to inhibit NF-kB and mTOR pathways by targeting the upstream central kinase IKKβ in pancreatic tumor and mantle cell lymphoma *in vitro* and *in vivo* [22, 23]. The dihydropteridinone derivative, small molecule inhibitor BI2536, has been shown to selectively inhibit mammalian PLK1 at low nanomolar concentrations in an ATP-competitive manner. In addition, BI2536 was well tolerated in phase I and II clinical trials, showing a favorable pharmacokinetic profile and antitumor activity in patients with various advanced solid tumors [24–26]. Vismodegib, a small molecule inhibitor of the hedgehog pathway, was developed by Genentech and is being investigated in clinical trials targeting several types of tumors [27–29]. In a search for novel strategies to target refractory neuroblastoma, we investigated the therapeutic efficacy of these small molecule inhibitors as single agents or in combination with chemotherapy against neuroblastoma.

In the present study, we have evaluated and screened the efficacy of the NF-kB/mTOR dual inhibitor 13-197, PLK1 inhibitor BI2536 and hedgehog pathway inhibitor vismodegib alone or in combination with topotecan against neuroblastoma. These small molecule inhibitors decreased the growth and induced apoptosis in neuroblastoma cells by targeting their pathways as single agents. Among these inhibitors, the hedgehog inhibitor not only showed single agent efficacy but also significantly enhanced antineuroblastoma efficacy of topotecan. Therefore, as a next logical step, we investigated the *in vivo* therapeutic efficacy of vismodegib in a neuroblastoma xenograft model and the results validated the *in vitro* outcome. Our data strongly support the continued development of vismodegib for the treatment of neuroblastoma.

## RESULTS

### Single agent efficacy of small molecule inhibitors on neuroblastoma cell growth and apoptosis

**Figure 1:**
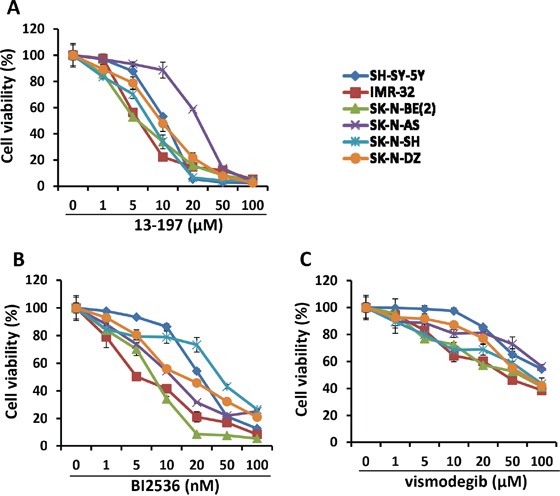
Effect of small molecule inhibitors on neuroblastoma cell growth *in vitro* Twenty thousand of each neuroblastoma cell line indicated were cultured in EMEM:DMEM-F12 complete media containing indicated concentration of 13-197 **A.** BI2536 **B.** and vismodegib **C.** or DMSO (vehicle, 0.05%)) in 96-well plates and the growth of these cells were determined at 72 hours using MTT assay. The values represent the means ± SD from four wells of 96-well plates.

**Figure 2:**
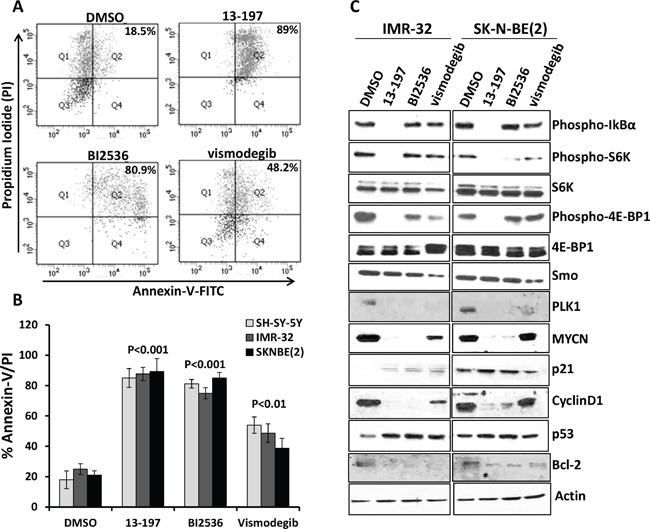
Single agent effects of small molecule inhibitors on the apoptosis induction and expression of their associated pathways/molecules in neuroblastoma cells Each neuroblastoma cell line was treated individually with inhibitors for 72 hours. SH-SY-5Y cells were treated with 13-197, BI2536 and vismodegib at 15 μM, 20 nM and 50 μM, respectively. Two other neuroblastoma cell lines including IMR-32 and SK-N-BE(2) were treated with 13-197, BI2536 and vismodegib at 10 μM, 10 nM and 50 μM, respectively. Following treatment, the percentage of cells undergoing apoptosis was determined using Annexin-V apoptosis detection assay. **A.** A representative scatter diagram for the apoptotic cell analyses following each inhibitor treatment in neuroblastoma cells. **B.** Quantification of the apoptotic cells (% Annexin-V/PI double positive) following treatment with each small molecule inhibitor in SH-SY-5Y, IMR-32 and SK-N-BE(2) neuroblastoma cells. The values represent the means ± SD of three separate experiments. **C.** IMR-32 and SK-N-BE(2) neuroblastoma cells were treated individually with 10 μM 13-197, 10 nM BI2536 and 50 μM vismodegib or DMSO (vehicle) for 24 hours and the expression of associated pathways/molecules were determined using western blot analyses. β-Actin was used as a loading control in these experiments.

In order to examine the efficacy of the small molecule inhibitors 13-197 (NF-kB/mTOR dual inhibitor), BI2536 (PLK1 inhibitor) and vismodegib (hedgehog inhibitor) on the proliferation and survival of neuroblastoma cells *in vitro*, three non-MYCN amplified (SH-SY-5Y, SK-N-SH and SK-N-AS) and three MYCN amplified (IMR-32, SK-N-BE(2) and SK-N-DZ) neuroblastoma cell lines were used. The concentrations used for those inhibitors were chosen from our observations and published literature and were in clinical achievable ranges [16, 22, 30–35]. The MTT results shown in Figure 1 clearly demonstrated a dose-dependent growth inhibition of neuroblastoma cells following treatment with all three inhibitors. 13-197 and BI2536 showed a strong growth inhibition and were most potent with IC_50_ values ranging 7.5 to 22.7 μM and 8.5 to 32.7 nM, respectively (Table 1). Compared to 13-197 and BI2536, vismodegib was found to be less effective with IC_50_ >50 μM in inhibiting the neuroblastoma cell growth. The inhibitors 13-197, BI2536 and vismodegib showed cell growth inhibition with IC_50_ of 8.5-22.7 μM, 13-32.7 nM and 73.6-85.4 μM, against non-MYCN amplified neuroblastoma cells, respectively. However, 13-197, BI2536 and vismodegib were able to suppress cell growth at IC_50_ of 7.5-9.1 μM, 8.5-15.5 nM and 61.3-75.8 μM against MYCN-amplified neuroblastoma cells, respectively. Together, these results demonstrated higher sensitivity of MYCN amplified neuroblastoma cells to these agents as IC_50s_ of all these inhibitors shifted upward in non-MYCN amplified cells. We next investigated the ability of these inhibitors to induce apoptosis in neuroblastoma cells following treatment at optimum concentrations for 72 hours. Our results of apoptosis analyses using Annexin-V assay clearly showed a significant induction of apoptosis in 13-197, BI2536 and vismodegib-treated neuroblastoma cells (Figures 2A and 2B). As shown in Figure 2B, treatment with 13-197 and BI2536 significantly induced (∼fourfold) apoptosis in all three neuroblastoma cell lines. Although, vismodegib induced apoptosis significantly (∼twofold), it was less effective in inducing apoptosis compared to 13-197 and BI2536. These results were consistent with growth studies (Figure 1) confirming the ability of these agents to inhibit growth of multiple neuroblastoma cell lines. Together, these results demonstrate that these small molecule inhibitors, as single agents, suppress the growth and survival of aggressive neuroblastoma *in vitro*.

**Table 1:** IC50 Values for small molecule inhibitors and topotecan in neuroblastoma cell lines

Cell Line/Agent	13-197 (μM)	BI2536 (nM)	vismodegib (μM)	topotecan (nM)
**SH-SY-5Y**	12.43	18.15	76.53	7.5
**IMR-32**	7.53	9.7	61.36	12.7
**SK-N-BE(2)**	8.2	8.56	75.85	60
**SK-N-AS**	22.74	13	85.46	10
**SK-N-SH**	8.52	32.75	73.65	8.54
**SK-N-DZ**	9.13	15.55	72.21	>100

IC50s of small molecule inhibitors and topotecan were calculated by non-linear regression analysis using GraphPad Prism software version 6.0.

### Small molecule inhibitors abrogate their associated pathways/molecules in neuroblastoma cells

We next investigated the molecular mechanism(s) of the small molecule inhibitor-mediated antineuroblastoma effects. We determined the expression/activation of inhibitor specific pathways/molecules following treatment with each inhibitor in IMR-32 and SK-N-BE(2) neuroblastoma cell lines. Our western blot results (Figure 2C) demonstrated that 13-197, a dual NF-kB/mTOR inhibitor, significantly downregulated the phosphorylated levels of IkBα (an upstream kinase of the NF-kB pathway) and mTOR pathway molecules such as S6K and 4E-BP1, and showed strong specificity to the NF-kB and mTOR signaling pathways. On the other hand, PLK1 inhibitor BI2536 and hedgehog inhibitor vismodegib were not able to modulate the levels of phosphorylated IkB of the NF-kB pathway. However, these inhibitors decreased the levels of phosphorylated S6K and 4E-BP1 molecules of the mTOR pathway compared to vehicle treated neuroblastoma cell lines, suggesting that BI2536 and vismodegib can also target the mTOR signaling pathway. We next determined the expression levels of smoothen (Smo) molecule of the hedgehog pathway in inhibitors-treated neuroblastoma cells. As expected, vismodegib significantly decreased Smo expression. However, 13-197 and BI2536 did not induce a significant decrease in Smo expression. We also examined the effect of inhibitors on PLK1 expression. BI2536 treatment resulted in complete shut-down of PLK1 expression in neuroblastoma cells. Similar effects were also observed for vismodegib and 13-197. Since PLK1 is a cell cycle regulator and downstream molecule that can be regulated by various oncogenic pathways, inhibitors of different pathways exhibited efficacy targeting PLK1. Further, all three inhibitors downregulated the expression levels of downstream molecules such as MYCN, Bcl-2 (apoptosis), cyclin D1 (proliferation), and upregulated the expression of tumor suppressor proteins such as p21 (cell cycle) and p53 (apoptosis) which are also known to be regulated by oncogenic pathways. Overall, these results suggest that all three inhibitors target their associated pathways and downstream molecules, thereby decreasing cell growth and inducing apoptosis in neuroblastoma cells.

### Combined effects of small molecule inhibitors with chemotherapy on neuroblastoma cell growth and apoptosis

**Figure 3:**
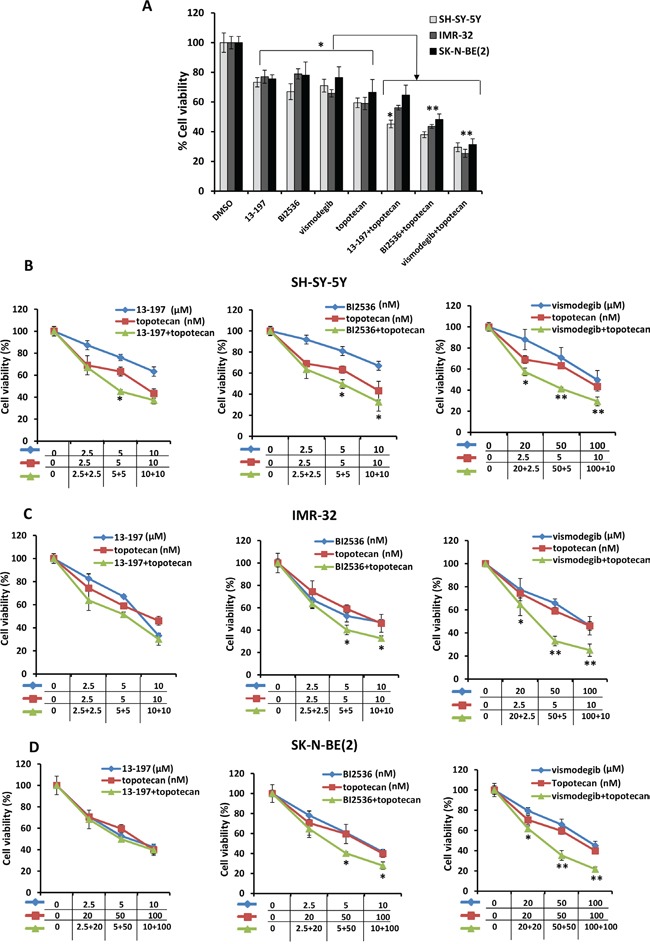
Effects of small molecule inhibitors alone or in combination with chemotherapy on neuroblastoma cell growth **A.** Shows a representative bar graph for the combination effects of inhibitors and topotecan on neuroblastoma cells growth treated with fixed one concentration of each inhibitor at sub-IC50 concentration for 72 hours. For combination treatments, SH-SY-5Y cells were treated with 13-197, BI2536, vismodegib and topotecan at 5 μM, 5 nM, 50 μM, and 5 nM concentrations, respectively; IMR-32 cells were treated with 13-197, BI2536, vismodegib and topotecan at 5 μM, 5 nM, 50 μM, and 10 nM concentrations, respectively; and SK-N-BE(2) were treated with 13-197, BI2536, vismodegib and topotecan at 5 μM, 5 nM, 50 μM and 50 nM concentrations, respectively. Following treatment, the cells were subjected to growth analyses using MTT assay. **B-D.** MTT assay showing the combination effects of indicated small molecule inhibitors and topotecan on neuroblastoma cells growth at 72 hours in a dose-dependent manner. The values represent the means ± SD from four wells of 96-well plates. *, p<0.05.

In order to investigate the combined effects of small molecule inhibitors with chemotherapy, we chose a topoisomerase 1 inhibitor topotecan which is used in the treatment of neuroblastoma. We first determined the IC_50_ of topotecan inhibition of neuroblastoma cell growth. The IC_50_ of topotecan was determined in non-MYCN and MYCN amplified neuroblastoma cell lines (Supplementary Fig. S1 and Table 1). The increased IC_50_ values of topotecan in MYCN-amplified cells (SK-N-BE(2), SK-N-DZ) indicated chemoresistance in these cells. For combination therapy, the neuroblastoma cell lines (SH-SY-5Y, IMR-32 and SK-N-BE2) were pre-exposed to inhibitors for 24 hours followed by topotecan at sub-IC_50_ concentrations for an additional 48 hours and then neuroblastoma cell growth and apoptosis were assessed. The representative MTT results shown in Figure 3A clearly showed that all three inhibitors and topotecan as single agents significantly decreased the cell growth of all neuroblastoma cell lines. However, the combined efficacy of the inhibitors with topotecan chemotherapy varied among cell lines. The combined efficacy of 13-197 with topotecan was found to be effective in decreasing growth significantly in SH-SY-5Y neuroblastoma cells, but not in IMR-32, SK-N-BE(2) cells. Combined treatment of BI2536 and topotecan showed significantly decreased the cell growth of all neuroblastoma cell lines. Interestingly, hedgehog pathway inhibitor vismodegib combined with topotecan exhibited higher efficacy in decreasing cell growth in all neuroblastoma cell lines when compared with BI2536 and 13-197 combinations (Figure 3A). Together, these results demonstrated a lower efficacy for 13-197, moderate efficacy for BI2536 and high efficacy for vismodegib when topotecan was combined with these inhibitors. These efficacies were further confirmed using soft-agar colony formation assay and results for their ability to inhibit colony formation were consistent with MTT cell growth results (Supplementary Fig. S2).

**Figure 4:**
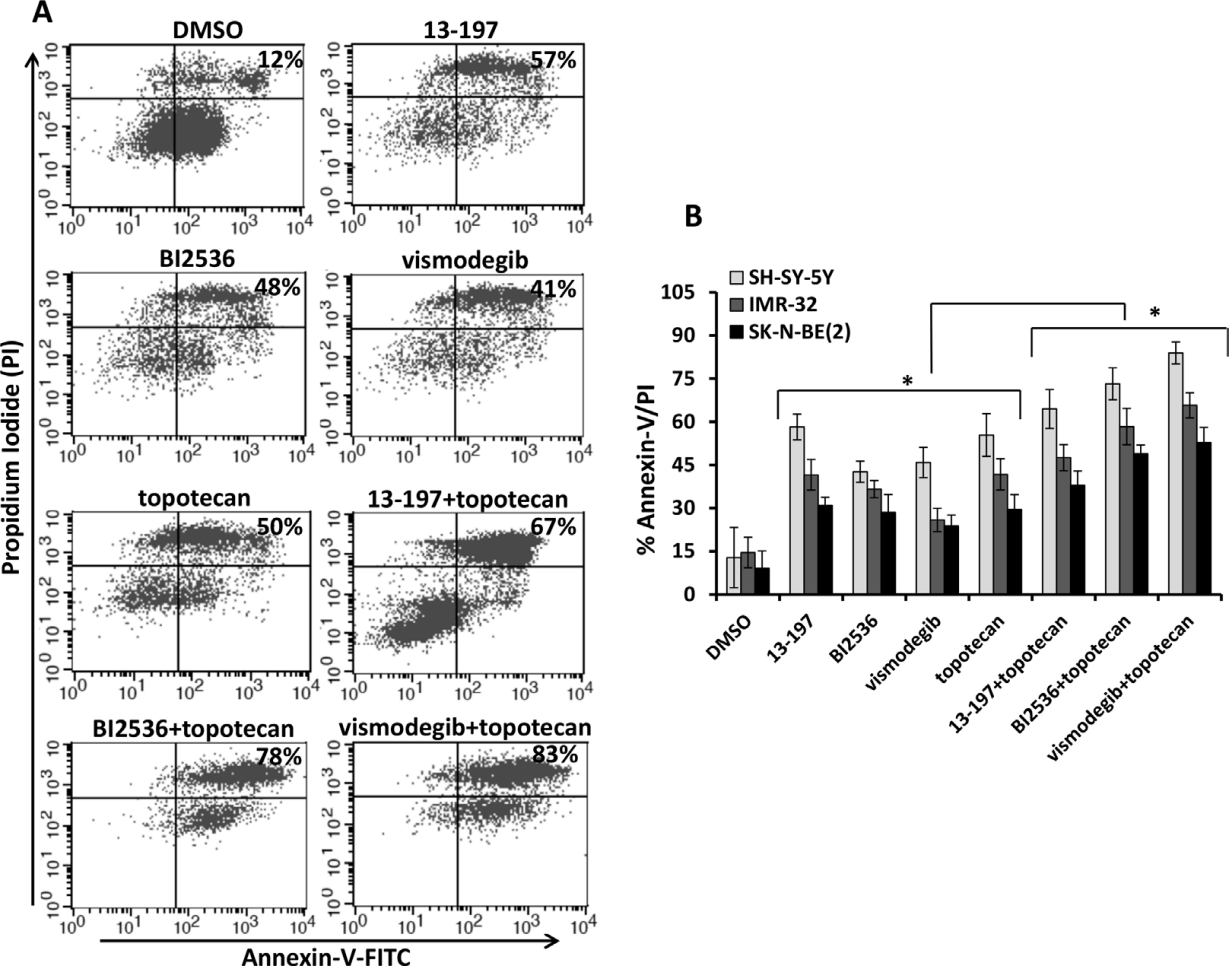
Effects of small molecule inhibitors alone or in combination with chemotherapy on neuroblastoma apoptosis **A.** Illustrates a representative scatter diagram for the apoptotic cell analyses following treatment as indicated above in Figure 3A with small molecule inhibitors alone or in combination with topotecan in neuroblastoma cells. **B.** Quantification of the apoptotic cells (% Annexin-V/PI double positive) following small molecule inhibitors alone or in combination with topotecan treatment in SH-SY-5Y, IMR-32 and SK-N-BE(2) neuroblastoma cells. The values represent the means ± SD of three separate experiments. *, p<0.05.

We also evaluated the combination efficacies of these inhibitors and topotecan at their fixed ratio with increasing concentrations. Our MTT results for their dose-dependent combination efficacies shown in Figure 3B-3D, showed similar efficacy in a dose-dependent fashion and were consistent with our previous observation. Overall, calculation of combination index (CI) confirmed that there was no synergistic/additive effect of 13-197 and topotecan combination. However, BI2536 and vismodegib combined with topotecan clearly demonstrated the additive and synergistic interaction, respectively (Table 2). These data further confirmed that the combination of vismodegib and topotecan was most efficacious against neuroblastoma even though vismodegib alone was not most effective single agent *in vitro*. We further tested the effects of these combinations in inducing apoptosis in neuroblastoma cells. Using Annexin-V assay, the results of these analyses showed significantly increased apoptosis by all inhibitors alone (Figures 4A and 4B). In combination with topotecan, all three inhibitors further increased apoptosis significantly in all neuroblastoma cell lines compared with the respective single agents (Figure 4B). The combination efficacy of these inhibitors in inducing apoptosis, were consistent with the growth inhibitory effects.

**Table 2:** Combination indexes of small molecule inhibitors and topotecan in neuroblastoma cell lines

SH-SY-5Y			IMR-32			SK-N-BE(2)		
13-197 (μM)	topotecan (nM)	CI	13-197 (μM)	topotecan (nM)	CI	13-197 (μM)	topotecan (nM)	CI
2.5	2.5	1.114	2.5	2.5	1.172	2.5	20	1.387
5	5	0.858	5	5	1.079	5	50	1.108
10	10	1.083	10	10	0.981	10	100	1.152
**BI2536** **(μM)**	**topotecan** **(nM)**	**CI**	**BI2536** **(μM)**	**topotecan** **(nM)**	**CI**	**BI2536** **(μM)**	**topotecan** **(nM)**	**CI**
2.5	2.5	1.042	2.5	2.5	1.126	2.5	20	0.951
5	5	0.824	5	5	0.834	5	50	0.727
10	10	0.853	10	10	0.758	10	100	0.815
**vismodegib** **(μM)**	**topotecan** **(nM)**	**CI**	**vismodegib** **(μM)**	**topotecan** **(nM)**	**CI**	**vismodegib** **(μM)**	**topotecan** **(nM)**	**CI**
20	2.5	0.821	20	2.5	0.823	20	20	0.826
50	5	0.553	50	5	0.485	50	50	0.564
100	10	0.758	100	10	0.574	100	100	0.585

Neuroblastoma cells were treated for 72 hours with indicated concentrations of inhibitors. Cell growth was determined Using MTT assay. CI was calculated by CalcuSyn software as described in Materials and methods for data shown in Figure 3.

### Effects of inhibitors and topotecan combination on associated pathways/molecules

**Figure 5:**
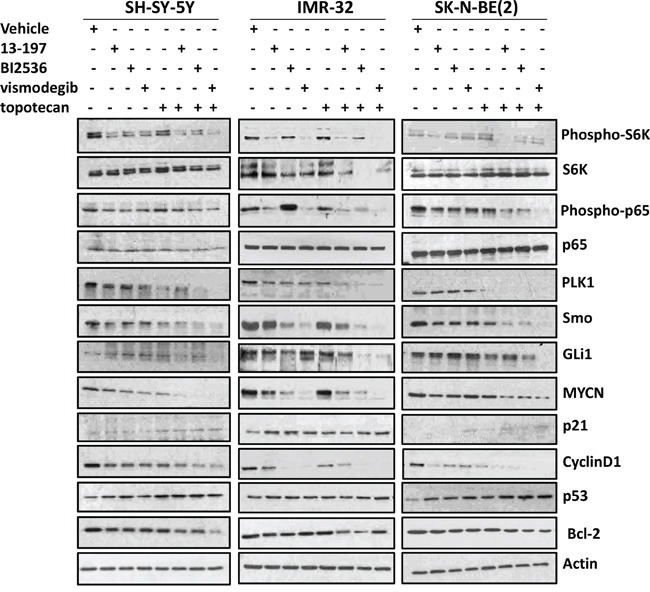
Effects of small molecule inhibitors alone or in combination with chemotherapy on the expression of associated pathways/molecules in neuroblastoma cells Each neuroblastoma cell line was treated with inhibitors alone or combination with topotecan (as described in Figure 3A) for 24 hours and the expression of associated pathways/molecules were determined using western blot analyses. β-Actin was used as a loading control in these experiments.

We next wanted to see whether combining these inhibitors of the NF-κB/mTOR, hedgehog and PLK1 with topotecan resulted in a corresponding declines in expression/activation of the respective pathways/molecules in neuroblastoma cells. We evaluated the combination efficacy of inhibitors on the expression of these target pathways/molecules in three neuroblastoma cell lines [SH-SY-5Y, IMR-32, and SK-N-(BE)2] using western blot analyses. Western blot data (shown in Figure 5) clearly showed that all inhibitors as single agents, significantly downregulated the levels of phosphorylated p65 (NF-kB) and S6K (mTOR) molecules in all three neuroblastoma cells. Topotecan alone did not show any effects on the expression of these molecules. In combination with topotecan, the expression levels of the phosphorylated p65 and S6K were further decreased in MYCN-amplified cells. However, there was no significant change observed on the expression of these molecules in non-MYCN amplified neuroblastoma cells, suggesting combination specificity to MYCN-amplified neuroblastoma cells targeting NF-kB and mTOR pathways.

We next tested the effect of these agents on the expression of PLK1 and hedgehog pathway molecules. Although the expression levels of PLK1 and Smo (hedgehog) varied among treatments and cell lines, their expression levels were only slightly decreased in individual agents-treated cells, but the expression in combination with chemotherapy was decreased significantly below detectable levels in all three neuroblastoma cell lines tested. The expression of transcription factor GLi1 of the hedgehog pathway in neuroblastoma cells treated with inhibitors and topotecan alone showed no significant changes. However, as expected, vismodegib combined with topotecan strongly decreased GLi1 expression and these results were consistent in all neuroblastoma cell lines.

Further, we evaluated the combination efficacy of inhibitors and topotecan on the expression levels of the downstream targets such as MYCN, cyclin D1, p21, Bcl-2 and p53 which are known to regulate cell proliferation and apoptosis. Our results showed that the inhibitors alone or in combination with chemotherapy significantly downregulated the expression of MYCN, Cyclin D1 and Bcl-2 molecules and upregulated the expression of tumor-suppressor proteins p21 and p53 in all three neuroblastoma cell lines. Interestingly, the expression levels of these molecules were further either decreased or increased respectively when the inhibitors were combined with topotecan. Together, these results demonstrate that these inhibitors exhibit potential to promote chemosensitivity in neuroblastoma cells. Also, comparing combination efficacies of the inhibitors and chemotherapy indicated a lower efficacy for 13-197, moderate efficacy for BI2536 and highest efficacy for vismodegib in modulating pathways/molecules. Overall, these results suggest that small molecule inhibitors combined with chemotherapy significantly decreased neuroblastoma cell growth and induced apoptosis mediated, at least in part, via targeting their associated pathways/molecules and vismodegib combined with topotecan was most effective.

### Combination efficacy of small molecule inhibitors and topotecan on neurosphere formation

**Figure 6:**
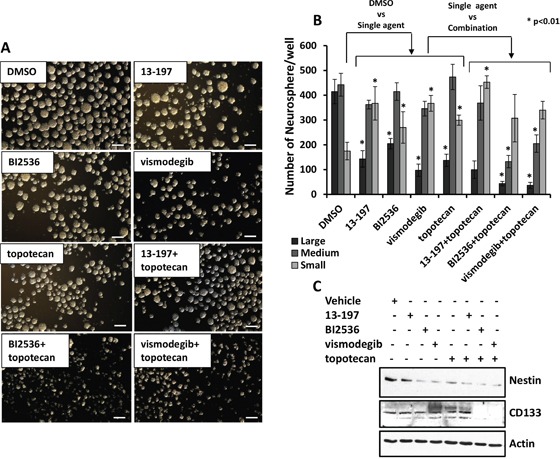
Antineurosphere efficacy of the molecule inhibitors alone or in combination with chemotherapy **A.** Shows a representative micrograph of small, medium and large neurospheres in 13-197 (5 μM), BI2536 (5 nM) and vismodegib (50 μM) alone or in combination with topotecan (10 nM) treated IMR-32cells. The micrographs were taken using a phase contrast microscope at 4X magnification. Scale bar; 100 μm. **B.** Quantification of sphere assay data in above described treated cells. The measurement of 50 to <100 μm size for small, >100 to 200 μm for medium and >200 μm size for the large neurosphere were considered. The values represent the means ± SD from three wells of 6-well plates. *, p<0.01. **C.** Western blot analyses for the Nestin and CD133 expression following the indicated treatments of neurospheres. β-Actin was used as a loading control in this experiment.

Neuroblastoma cell lines contain tumor initiating cells which express stem cell markers such as Nestin and CD133 [36]. Neurospheres may be further enriched for cancer stem cells and these stem cells may represent potential targets for therapy-resistance [36–39]. Therefore, we next determined the efficacy of inhibitors alone or combined with chemotherapy on neurosphere formation in IMR-32 cells. Figure 6A shows a representative micrograph for small, medium and large sizes of neurospheres in agent-treated cells. We found that inhibitors and topotecan as single agents decreased the numbers of large size neurospheres significantly and significantly increased the numbers of small size neurospheres when compared to vehicle treated cells (Figures 6A and 6B). Interestingly, compared to their efficacy as single agents, BI2536 or vismodegib combined with topotecan induced a significant reduction in large and medium sizes of neurospheres, as well as total numbers of neurospheres (Figure 6B), indicating potency of these agents to shift large spheres to small spheres by inhibiting neurosphere formation. Nevertheless, we did not observe a significant effect of 13-197 combined with topotecan on numbers and sizes of the neurospheres compared to single agent effects (Figure 6B). We further tested the efficacy of these agents to modulate expression of neural stem cells markers (Nestin and CD133) in lysed neurosphere by western blotting. Western blot results shown in Figure 6C demonstrated that inhibitors and topotecan as single agents markedly decreased the expression of Nestin. The results also clearly demonstrated a significantly further decreased expression of Nestin and CD133 when inhibitors were combined with topotecan. Together, these data suggested that small molecule inhibitors alone or combined with chemotherapy targeted molecules associated with tumorigenic (cancer stem) cells thereby inhibiting neurosphere formation.

### Combination efficacy of vismodegib and topotecan in a xenograft mouse model *in vivo*

**Figure 7:**
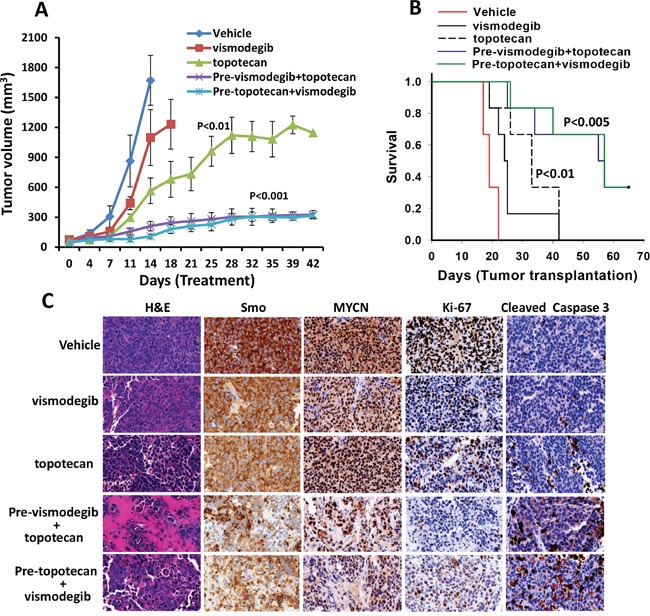
Combined antineuroblastoma efficacy of the hedgehog pathway inhibitor and chemotherapy in SK-N-BE(2) xenografts in mice NSG mice bearing tumors were treated with vehicle (DMSO) or vismodegib (50 mg/kg) and topotecan (10 mg/kg) alone, or both, twice a week for four weeks. For the combination, mice were treated with each agent first (denoted as Pre-) and then next day with the other agent. **A.** Tumor growth analyses following treatments. **B.** Kaplan–Meier analyses for the survival of mice using the log-rank test. **C.** Histological (H&E) and immunohistochemical (Smo, Ki-67, MYCN and cleaved caspase3) analyses of tumors in mice treated as indicated. The images were scanned and captured using a digital scanner VENTANA Image software (Roche, Germany) at ×40 magnification.

In our *in vitro* studies, we found that the combination of hedgehog inhibitor vismodegib and topotecan had the highest antineuroblastoma efficacy. Therefore, to validate the *in vitro* results, we further tested the antineuroblastoma efficacy of combination of vismodegib and topotecan in NSG mice bearing aggressive MYCN-amplified SK-N-BE(2) neuroblastoma cells. The tumor bearing mice were treated with vismodegib and topotecan alone or in combination. A week after treatment started, in vehicle treated control mice, the tumors grew rapidly and reached a tumor size 1000 mm^3^ of exceeding (Figure 7A). Consequently, as per protocol, these mice were euthanized 10 to 17 days post treatment. Vismodegib alone had significant effects (p<0.05) in reducing tumor burden but did not delay prolonged tumor growth. However, topotecan alone treated mice showed a significant (p<0.01) decrease in tumor burden compared to vehicle controls and vismodegib treated mice (Figure 7A). Vismodegib combined with topotecan, highly significantly (p<0.001) reduced the tumor growth compared to controls and tumor size was maintained at near baseline tumor volumes (Figure 7A, Supplementary Fig. S3-A). We next determined the survival of these treated mice. A maximum 1000 mm^3^ tumor size was set as end point for the survival analyses. The survival data clearly demonstrated that xenografted mice treated with combination of vismodegib and topotecan exhibited a highly significantly increased survival when compared to mice treated with vismodegib and topotecan alone (Figure 7B). These results were consistent with *in vitro* tumor growth studies. To evaluate the overall toxicity of the combination, we measured the body weight of these mice during the treatments. There was no significant difference in body weights between control and treatment groups (See Supplementary Fig. S3-B), suggesting the tolerability of the combination. Further, we determined the effect of vismodegib alone or in combination with topotecan on the expression of Smo (hedgehog pathway), MYCN, Ki-67 (proliferation) and cleaved caspase3 (apoptosis) molecules in xenografted tumors. Immunohistochemistry, (Figure 7C) clearly indicated that the combination of vismodegib and topotecan significantly decreased the expression of Smo, MYCN and Ki-67, and increased the levels of cleaved (activated) caspase 3, suggesting vismodegib combined with topotecan not only enhanced the reduction in tumor growth and increased survival, but also targeted the associated pathways and downstream molecules in xenograft tumors. Therefore, we concluded that combination therapy of vismodegib and topotecan induced a significant reduction of tumor growth and increased survival compared to use of the individual drugs alone. This confirmed that the small molecule inhibitor vismodegib potentiated the effects of topotecan, via targeting of the hedgehog pathway as an integrated mechanism together with topoisomerase-1 inhibition in the treatment of MYCN amplified, therapy-resistant neuroblastoma.

## DISCUSSION

Current therapy for high-risk neuroblastoma includes a multimodal approach utilizing chemotherapeutic agents including topotecan which produces good overall response rates (60%) but at the expense of significant toxicity including myelosuppression and subsequent infection [1–3]. Therefore, the combination of topotecan with strategies targeting oncogenic pathways and molecules has increasingly become important for improving outcomes in neuroblastoma and other cancers [40]. In addition, overexpression and activation of the key cellular pathways such as NF-kB, mTOR, hedgehog and PLK1 molecules are associated with resistance to therapy in neuroblastoma [15–19]. Targeting these pathways and molecules using small molecule inhibitors that have lower toxicity and have molecular target specificity and also enhance cytotoxic chemotherapy may provide improved treatment outcomes in therapy-resistant neuroblastoma.

Therefore, in the present study, we have investigated the therapeutic efficacy of small molecule inhibitors 13-197, NF-kB and mTOR pathways dual inhibitor; vismodegib, hedgehog pathway inhibitor and BI2536, PLK1 inhibitor either alone or in combination with topotecan against non-MYCN and MYCN-amplified neuroblastoma cell lines. These inhibitors not only showed single agent activity against neuroblastoma but also demonstrated significant enhancement of activity in combination with topotecan *in vitro*. Among these inhibitors, vismodegib combined with topotecan demonstrated the highest activity. Therefore, as the next logical step we examined the efficacy of vismodegib combined with topotecan *in vivo* using a mouse xenograft model. NSG mice transplanted with MYCN amplified tumor showed a significantly decreased tumor burden following combined treatment with vismodegib and topotecan. Vismodegib combined with topotecan not only significantly decreased the tumor growth but also exhibited very significant increase in survival of the xenografted mice without significant toxicities. In addition, this combination demonstrated decreased expression levels of hedgehog pathway molecules and associated downstream molecules, including MYCN, in xenografted tumors (Figure 7).

Since MYCN gene amplification occurs in about 50% of high-risk neuroblastoma patients [4], we used three MYCN-amplified and three non-MYCN-amplified neuroblastoma cell lines in present study. Our findings in the three MYCN amplified cell lines clearly indicated that MYCN-amplified (IMR-32, SK-N-BE(2), SK-N-DZ) cells shows greater sensitivity to small molecule inhibitors and lower sensitivity toward chemotherapy as IC_50_ values shifted lower for inhibitors and higher for topotecan in these cells compared to non-MYCN-amplified (SH-SY-5Y, SK-N-SH, SK-N-AS) cells (Figure 1 and Table 1). In addition, we have found that inhibitors combined with topotecan significantly decreased expression levels of phosphorylated p65 (NF-kB) and S6K (mTOR) pathways molecules in MYCN-amplified cells but not in non-MYCN-amplified neuroblastoma cells, indicating further activity of inhibitors in aggressive MYCN-amplified cells (Figure 5). However, topotecan alone did not show any changes in the expression of these molecules, suggesting that NF-kB/mTOR pathways play roles in chemoresistance and inhibitors combined with chemotherapy increased chemosensitivity in MYCN-amplified neuroblastoma cells. Thus these results are significant in promoting targeted-therapy using small molecule inhibitors for therapy-resistant neuroblastoma.

Molecularly, all three inhibitors as single agents, significantly downregulated the expression of NF-kB/mTOR hedgehog and PLK1 Pathways/molecules and downstream molecules. In addition, these inhibitors in combination with topotecan further enhanced the therapeutic efficacy in abrogating corresponding pathways/molecules in neuroblastoma cells. Based on our analyses of the results, the significant increase in therapeutic efficacy of the combination of these two drugs appears due to synergistic effects. Furthermore, the combination might have induced additional sensitivity to the pathways/molecules. Since signaling pathways that control cell growth and survival mechanisms are interactive and often involved in cross-talk with each other in various types of human cancers [41–43], a specific pathway/molecule inhibitor can also inhibit off-target pathways. Therefore, our findings with targeting pathways and molecules demonstrated that each inhibitor targeted their specific pathway, as well as off-target pathways and molecules. The understanding of off-target mechanism(s) by pathways specific inhibitors needs further investigation since it might have implications for toxicities. Overall, our molecular analyses of these inhibitors demonstrate their efficacy in inhibiting neuroblastoma cell growth and improving survival by targeting associated pathways and their regulated downstream molecules.

Cancer stem cells or tumor initiating cells have been implicated in tumor relapse and resistant to chemotherapy in various malignancies including neuroblastoma [36]. Neuroblastoma cells express neural stem cell markers such as Nestin and CD133 that may relate to their ability to form neurospheres [37–39]. In the present study, we have evaluated the effects of small molecule inhibitors alone or in combination with chemotherapy on neurosphere formation and expression of neural stem cell markers (Nestin, CD133). Our results provide evidence that the inhibitors as single agents disrupt organized neurospheres and downregulate the expression of the neural stem cell marker Nestin and, in combination with topotecan, further enhanced efficacy of disrupting neurospheres and downregulating Nestin expression (Figure 6). Although inhibitors alone did not produce an alteration in the expression of CD133, another neural stem cell marker, the inhibitor in combination with topotecan, led to a reduction in CD133 expression. This indicates the value of combination therapy for the targeting of “cancer stem cells” with the potential of reducing recurrence of neuroblastoma, thus improving progression free survival.

In our *in vitro* and *in vivo* findings, we found that, vismodegib alone had the least effect against neuroblastoma. However, in combination with topotecan, it showed a greater efficacy against neuroblastoma both *in vitro* and *in vivo*. The precise mechanism of the greater efficacy of this combination is not yet known. However, it is possible that the combination might have greater efficacy in killing neuroblastoma stem cells with activated hedgehog pathway. Further investigation is required to define the complete mechanisms of efficacy of this inhibitor. In this study, we have used topotecan at 10 mg/kg which is similar intraperitoneal dose used earlier in an ovarian cancer preclinical model [32]. They showed that there was no significant toxicity associated with this dose [31–32]. However, in reporting a pilot study, Park et al recommend using higher dose of topotecan to ensure needed therapeutic efficacy for newly diagnosed high risk neuroblastoma patients [33]. Moreover, we did not observe any significant changes in the body weight which indicated no overall toxicity between control and drug plus topotecan treated mice (Supplementary Fig. S3-B).

In summary, the combination of small molecule inhibitors of the NF-kB, hedgehog and PLK1 with topotecan demonstrated significant preclinical efficacy against neuroblastoma. In these combinations, the hedgehog pathway inhibitor vismodegib combined with chemotherapy showed the greatest increased efficacy both *in vitro* and *in vivo*. The findings from this study warrants further preclinical evaluation of this approach, including a full toxicology study of vismodegib and topotecan used in combination, in order to translate these observations to the clinic.

## MATERIALS AND METHODS

### Cell lines and maintenance

The human neuroblastoma cell lines MYCN non-amplified (SH-SY-5Y, SK-N-SH, SK-N-AS) and MYCN amplified (IMR-32, SK-N-BE(2), SK-N-DZ) were obtained from American Type Culture Collection (ATCC). These cell lines were authenticated by ATCC. These cell lines were cultured in EMEM and DMEM-F12 mixed (1:1 ratio) media (ATCC) containing 10% FBS, 1% penicillin, and 1% streptomycin (Invitrogen, CA). The cultures were maintained in a humidified incubator at 5% CO_2_ and 95% air atmosphere at 37°C. All cultures were passaged at 80-90% confluence.

### The therapeutic agents

The small molecule inhibitor 13-197 was provided by Dr. Amarnath Natarajan (University of Nebraska Medical Center). Hedgehog inhibitor vismodegib was purchased from LC laboratories (Woburn, MA) and PLK1 inhibitor BI2536 and chemotherapy topotecan were purchased from Selleckchem Company (Houston TX). These inhibitors were dissolved in DMSO at appropriate concentrations and stored at −20°C.

### *In vitro* growth assay

To determine the therapeutic efficacy of inhibitors, twenty thousand of each neuroblastoma cell line were plated and cultured in 200 μl complete media containing different concentrations of 13-197 (1-100 μM), vismodegib (1-100 μM), BI2536 (1-100 nM), topotecan (1-100 nM) or DMSO (vehicle) in 96-well plates and the growth of these cells were determined at 72 hours using a MTT assay. The IC_50_ values of the each drug for each cell line were determined using GraphPad Prism V6 software as described in Table 1. To determine the combined efficacy of the small molecule inhibitors with chemotherapy, the cells were pre-treated with inhibitors for 24 hours followed by topotecan for an additional 48 hours at sub-IC_50_ concentrations. Briefly, 25 μl of MTT reagent (5 mg/ml in PBS) was added to the cultures and incubated for 2 hours before the respective time point, and the cells were lysed using an SDS-based lysing reagent. The intensity of the color developed was determined at a 570 nm wavelength using a plate reader (Biotek, Germany).

### Colony forming assay

To determine the efficacy of inhibitors on colony forming ability against neuroblastoma cells, colony forming assays were performed using 0.3% agar semi-solid medium. Hundred thousand cells from each cell line were mixed with the aforementioned medium containing DMSO or inhibitors alone/or combined with topotecan and plated in triplicate in 6-well plates and incubated at 37°C with 5% CO_2_ for 2 weeks. Following incubation, aggregates larger than 50 cells were counted using an inverted microscope, as colonies.

### Apoptosis assay

The ability of inhibitors to induce apoptosis in neuroblastoma cell lines was determined using an Annexin-V:APC flow cytometry assay kit (BD Biosciences, CA) following the manufacturer's instructions. Briefly, 0.3 × 10^6^ cells/ml neuroblastoma cell lines were plated in 12-well plates and cultured and treated with inhibitors alone or in combination with topotecan for 72 hours. The percent of the cells undergoing apoptosis was then assessed using Annexin-V/propidium iodide double staining.

### Western blotting

Western blot analysis of the inhibitor-treated cells was performed using a standardized protocol [22]. The primary human antibodies used in these analyses included NF-κB (p65), Smo, p53, p21, Nestin and β-actin (Santacruz, CA), IκBα, phospho-IκBα, phospho-NF-κB (p65), Bcl-2, S6K, phospho-S6K, 4E-BP1, phospho-4E-BP1, PLK1 and N-Myc (Cell Signaling Technology, MA) and cyclin D1, Bcl-2, and CD133 (BD Biosciences, CA). Immunoreactivity was detected using appropriate peroxidase-conjugated secondary antibodies (Santacruz, CA) and visualized using an enhanced chemiluminescence (ECL) detection system (Pierce, IL).

### Neurosphere formation assay

IMR-32 neuroblastoma cells were seeded into 6-well plates at a density of 0.3 × 10^6^ cells/well and grown in neural stem cell media. Once neurospheres were established, the wells were treated with each inhibitor alone or in combination with topotecan chemotherapy as described earlier. After 72 hours, small (50 to <100 μm), medium (>100 to 200 μm) and large neurospheres (>200 μm) were observed under phase contrast microscope and quantitated. Sphere numbers were determined by manual counting, and the results were expressed as the mean sphere number of treated wells as compared with DMSO-treated wells. Neurosphere lysates from the different treatments were also subjected to western blot analyses for the expression of neural stem cell markers including Nestin and CD133.

### *In vivo* studies

All animal experiments were carried using a UNMC Institutional Animal Care and Use Committee (IACUC) approved protocol. For these studies, six- to eight-week-old NOD-SCID common gamma chain knockout (NSG) mice from Jackson Laboratories (Bar Harbor, ME) were injected subcutaneously in the flank with 1 × 10^6^ SK-N-BE(2) neuroblastoma cells suspended in 100 μl PBS and mixed 1:1 with matrigel (BD Biosciences). Eight days post-tumor injection, when tumor size was 50-100 mm^3^, the tumor bearing mice were divided into five treatment groups (n=6 per group) and treated twice a week for four weeks. Treatments included vehicle control (DMSO, i.p.), hedgehog pathway inhibitor vismodegib (50 mg/kg, i.p.) alone, topotecan chemotherapy (10 mg/kg, i.p.) alone and combination vismodegib (50 mg/kg, i.p.) + topotecan (10 mg/kg, i.p.) in two sequences. The doses used for these inhibitors were at ranges to achievable exposures in mice or humans [30–33]. For the combination treatments, the mice were first treated with vismodegib or topotecan on one day and the next day treated with other agent (based on *in vitro* data). Tumor growth was assessed twice a week using a digital caliper and tumor volume was quantitated using the formula (length × width2 × 0.5). When tumor volume approached 1,000 mm^3^, the mice were euthanized using CO_2_ and tumor tissues were collected and processed for the histological/immunohistological analyses. The survival of the vehicle or different agent–treated mice was determined by the Kaplan–Meier method and analyzed for statistical significance using the log rank test.

### Statistical analysis

Each experiment was performed in triplicate and repeated an additional 2–3 times and the mean and standard error values of all experiments calculated. The significance of differences (*p*-value) was calculated using independent Student t-tests and the *p*-values less than 0.05 considered significant. To determine inhibitor combinations/interactions, we used the Chou and Talalay CI method using CalcuSyn software (Biosoft, Cambridge, UK). CI<0.9 indicates synergism, 0.9-1.1 additivity and >1.1 antagonism.

## SUPPLEMENTARY FIGURES


