# Supplementary material for: Improved therapy for neuroblastoma using a combination approach: superior efficacy with vismodegib and topotecan
Source: Oncotarget. 2016 Feb 25;7(12):15215–29. doi: 10.18632/oncotarget.7714 (PMC4924781; doi:10.18632/oncotarget.7714)
Supplement: Supplementary file 1 [file oncotarget-07-15215-s001.pdf]

SUPPLEMENTARY FIGURES

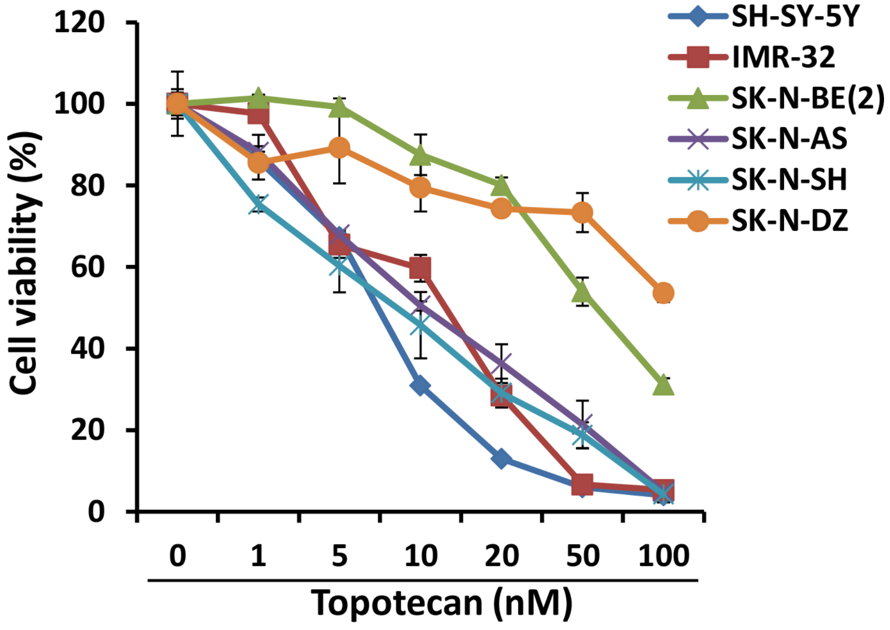

Supplementary Figure S1: Effect of topotecan on neuroblastoma cell growth. MTT assay showing the effect of topotecan on MYCN-amplified [IMR-32, SK-N-BE(2) and SK-N-DZ] and non MYCN-amplified (SH-SY-5Y, SK-N-AS and SK-N-SH) neuroblastoma cells growth in a dose-dependent manner.

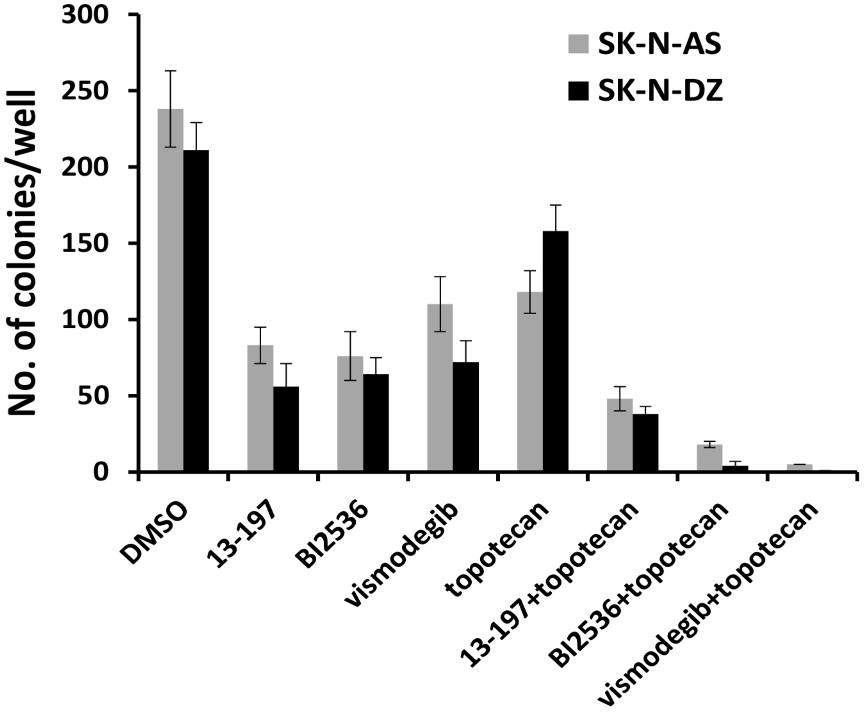

Supplementary Figure S2: Combination effects of small molecule inhibitors and topotecan on colony formation ability in non MYCN (SK-N-AS) and MYCN amplified (SK-N-DZ) neuroblastoma cells. Graph bars represent the average number of colonies from triplicate wells of 6-well plates.

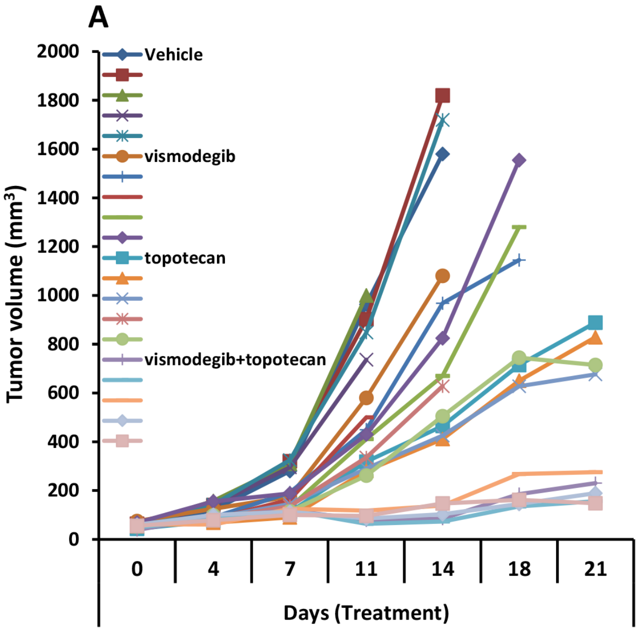

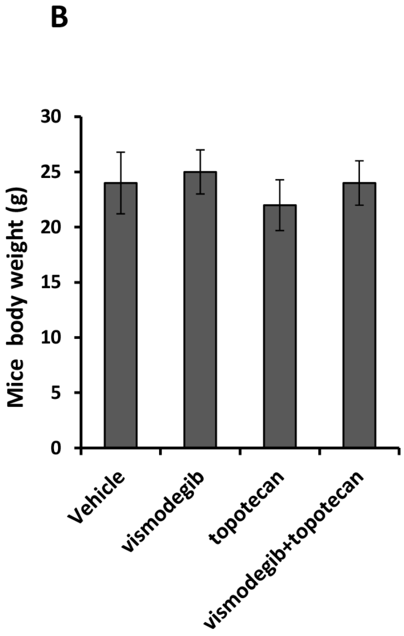

Supplementary Figure S3: Combination effect of vismodegib and topotecan on tumor growth and body weight of the xenograft mice. A. Showing the individual mouse tumor growth in indicated treatment groups. **B.** Shows the mean body weight of mice in control and treatment groups.
